# A Method for Pipeline Leak Detection Based on Acoustic Imaging and Deep Learning

**DOI:** 10.3390/s22041562

**Published:** 2022-02-17

**Authors:** Sajjad Ahmad, Zahoor Ahmad, Cheol-Hong Kim, Jong-Myon Kim

**Affiliations:** 1 Department of Electrical, Electronic and Computer Engineering, University of Ulsan, Ulsan 44610, Korea; sajjad907@mail.ulsan.ac.kr (S.A.); zahooruou@mail.ulsan.ac.kr (Z.A.); 2 School of Computer Science and Engineering, Soongsil University, Seoul 06978, Korea; cheolhong@ssu.ac.kr; 3 PD Technology Cooperation, Ulsan 44610, Korea

**Keywords:** acoustic emission signals, continuous wavelet transform, deep learning, leak detection

## Abstract

This paper proposes a reliable technique for pipeline leak detection using acoustic emission signals. The acoustic emission signal of a pipeline contains leak-related information. However, the noise in the signal often obscures the leak-related information, making traditional acoustic emission features, such as count and peaks, less effective. To obtain leak-related features, first, acoustic images were obtained from the time series acoustic emission signals using continuous wavelet transform. The acoustic images (AE images) were the wavelet scalograms that represent the time–frequency scales of the acoustic emission signal in the form of an image. The acoustic images carried enough information about the leak, as the leak-related information had a high-energy representation in the scalogram compared to the noise. To extract leak-related discriminant features from the acoustic images, they were provided as input into the convolutional autoencoder and convolutional neural network. The convolutional autoencoder extracts global features, while the convolutional neural network extracts local features. The local features represent changes in the energy at a finer level, whereas the global features are the overall characteristics of the acoustic signal in the acoustic image. The global and local features were merged into a single feature vector. To identify the pipeline leak state, the feature vector was fed into a shallow artificial neural network. The proposed method was validated by utilizing a data set obtained from the industrial pipeline testbed. The proposed algorithm yielded a high classification accuracy in detecting leaks under different leak sizes and fluid pressures.

## 1. Introduction

Pipelines play an important role in the distribution of liquid and gas resources. However, a leak in a pipeline can lead to severe consequences such as wasted resources, distribution downtime, risks to community health, and economic losses [1]. To avoid these consequences, early leak detection is important. Techniques, such as reflectometry in the time domain, vibration-based techniques, pressure wave techniques, and acoustic emission (AE) technology, have been proposed in the past for pipeline condition monitoring [2,3,4,5,6,7]. Due to the fact of their sensitivity to leaks and real-time leak detection response, AE technologies have received significant attention [8]. A significant amount of research has been conducted on pipeline leak detection. This research was based on vision, sensors, transient response, models, and data [9]. Each method of detecting leaks has its drawback, which prevents their use in leak detection. Some are time-consuming, and the rest are expensive. To address these issues, artificial intelligence (AI) comes into play. AI is fast, precise, and effective. AI readily enhances the process of automation in every field of life, aiding humans in performing tasks more efficiently and effectively. Hence, AI was utilized to detect leaks in this study.

In the past decade, researchers have focused on feature extraction and feature recognition models for leak detection in pipelines [10,11]. AE has been used for condition monitoring in many methods [12]. Elforjani et al. used AE technology for crack initiation detection [13]. Banjara et al. [14] detected pipeline leaks by utilizing AE waveform features, support vector machines (SVMs), and relevance vector machines. Rai et al. [15] developed a pipeline health index based on the Kolmogorov–Smirnov (KS) test and multiscale analysis. Furthermore, to determine the severity of the leak, a Gaussian mixture model was used. Kim et al. [16] developed a pipeline leak indicator by utilizing AE waveform features and a two-sample KS test. The study showed that the proposed leak indicator outperformed the traditional feature (i.e., mean, variance, and root mean square)-based leak indicators. Li et al. [17] combined the AE time-domain features with AE frequency domain features to form a hybrid feature vector. To enhance the accuracy of artificial neural networks (ANNs) for leak detection, the study selected discriminant features from the hybrid feature vector using cross-entropy. Xu et al. [18] used time–frequency methods, such as continuous wavelet transform and empirical mode decomposition (EMD), for pipeline leak location identification. Xu et al. [19] denoised the AE signal by using variational mode decomposition (VMD). Furthermore, the Mel frequency cepstral coefficients (MFCCs) were extracted as a feature from the highly correlated coefficients of VMD. To identify the condition of the pipeline, the MFCCs were classified using SVM. The leak recognition model could recognize leaks using AE features; however, the pre-defined threshold for extracting the AE features can lead to false alarms due to the fact of noise in the AE signal. Furthermore, extracting AE features from the AE signal requires human expertise and domain understanding. Using EMD for obtaining the intrinsic modes from the AE signal leads to extreme interpolation. Furthermore, mode mixing is also a challenge with EMD.

Deep learning (DL) methods can solve the problem of leak-related information extraction and classification [20]. DL algorithms enable feature extraction from the image without human intervention [21]. The issue of identifying transients due to the fact of unexpected leaks still hinders perfect leak detection. The DL methods extract features to identify them, but they are not 100% accurate. Among the DL methods, a CNN is the prominent technique. CNNs have proved to be vital in leak detection and fault diagnosis [22]. A CNN can extract leak-related discriminant information from acoustic images and can utilize it for pipeline state classification [23,24,25,26,27]. For intelligent fault detection, Jiao et al. used a residual joint adaptation adversarial network [28] and a deep coupled dense convolutional network [29], which are very interesting and are considered for future research on leak detection. Convolutional autoencoders (CAEs) can perform image compression and recognition of anomalies after learning representative information from the data provided at the input [30]. A change in the AE phenomenon increases the energy of the AE signal detected by the AE sensors in the form of hits. These hits can be overwhelmed by interference noises [31]. Continuous wavelet transform (CWT) can be used to analyze leak-related useful hits. CWT takes the time-domain signal and converts it to time–frequency scales [32]. These time–frequency scales result in a scalogram that can capture the hits in the AE signal over different time–frequency scales in the form of a 3D image.

A leak in the pipeline results in stress waves. AE sensors installed on the pipelines record these stress waves as they transmit through the pipeline walls. The leak-related stress waves produce transients in the AE signal known as hits or AE events. Therefore, traditional AE features, such as rise time, decay time, and counts, can be extracted from the AE signal by defining a threshold above the level of continuous background noise. As mentioned earlier, the pre-defined threshold for extracting AE features can lead to false alarms due to the fact of noise in the AE signal. Furthermore, extracting AE features from the AE signal and defining a threshold above the level of continuous background noise requires human expertise and domain understanding. To address this problem, this paper proposes a new deep learning-based model that extracts the leak-related features from the AE signal without human intervention. To utilize the leak-related transient in the AE signal, the proposed method converts the AE signal into AE images. Then, the proposed deep learning-based model extracts features from the AE images obtained from an experimental setup for pipeline leak detection. The identification of the leak against the normal signal is made based on the extracted features. Here, many convolutional filters of a CNN, which pass over the input scalograms to pick up the patterns in certain local parts, backed by kernels, are used to extract half of the feature pool, referred to as “local features” in this paper. At the same time, the latent space representations in the bottleneck layer of the trained convolutional autoencoder complete the second half of the feature pool, referred to as “global features” in this paper. These high-level features are the basis of input data that help the autoencoder to reconstruct the input scalogram. At the end, the feature pool with both the global and local features is used to assess the health of the pipeline through an ANN. The main contributions of this study were:

- The visualization and separation of the leak features from noisy data; the time-domain AE signals were transformed into AE images using CWT. From these AE images, leak-related features could be extracted. To surpass the traditional feature extraction techniques, a novel deep neural framework of CNN–CAE was proposed to extract the features autonomously. To the best of the author’s knowledge, CNN–CAE-based leak-related feature extraction has not been presented in the literature so far;
- The feature space obtained from the CNN–CAE was classified into “leak” and “no leak” states of the pipeline under variable leak and pressure conditions using a shallow ANN;
- Real-world pipeline data were used for the validation of the proposed method.

The proposed method for leak detection was applied to a metallic steel pipe. Some studies suggest that leak detection in plastic pipes is challenging [33,34]. In the future, we will apply the proposed technique to improve leak detection in plastic pipes.

The remaining part of this study is organized as follows: the proposed method is presented in Section 2. Section 3 describes the pipeline leak experimental setup and data collection. The results obtained from the proposed method are explained in Section 4. Section 5 summarizes the conclusions of this study and provides future research direction.

## 2. Proposed Method

**Figure 1:**
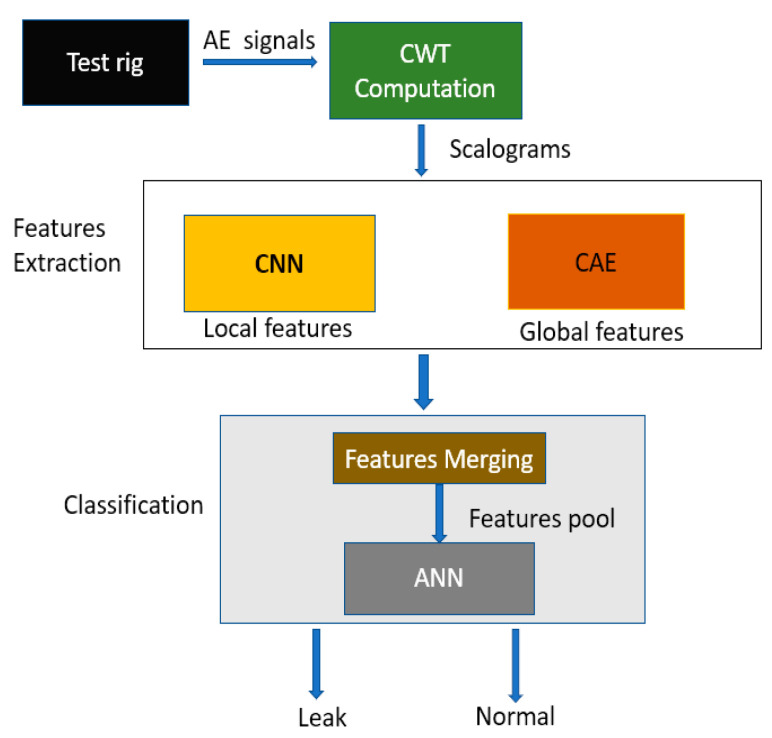
Proposed methodology for leak detection.

The overall flow of the proposed method is illustrated in Figure 1. The steps involved in the proposed method are as follows:

Step 1: AE signals are collected from the pipeline testbed using AE sensors;

Step 2: AE images are obtained from the AE signal by transforming it using CWT. The CWT scalograms obtained from the AE signal represent the time-domain AE signal over different time–frequency scales in a 3D image, referred to as AE images in this paper. The change in the color intensities in the AE images shows the change in energy over different time–frequency scales;

Step 3: To extract leak-related discriminant features from the AE images, the images are provided as inputs to the CAE and CNN to extract global features and local features, respectively. The local features represent the change in the energy at a finer level, and the global features are the overall acoustic signal characteristics in the AE image; 

Step 4: The global and local features are merged into a single feature vector. The single feature vector is provided as an input to the ANN for pipeline leak state identification under different leak sizes and pressures.

The above steps are performed using CWT, CAE, CNN, and ANN, which are described in greater detail below.

### 2.1. Continuous Wavelet Transform-Based Acoustic Emission Images

A CWT transforms a time-domain complex signal into the time–frequency domain with the help of the source wavelet function. The source or mother wavelet is usually a short time-based signal such as a vibrating signal having cycles [20]. The mother wavelet is the basis for decomposition, and a complex signal is decomposed into coefficients localized with translation and scale parameters.

When applied to a real-valued pipeline AE signal, CWT results in a 2D transformation matrix. Each row in the transformed matrix represents one scale of the AE signal obtained from the pipeline, while the columns represent the translation or the pipeline AE signal size. Thus, these 2D transformation matrices can be represented in the form of an image called an AE scalogram. The color intensities of the scalogram show the maximum and minimum energy spreads of the wavelet in time and frequency according to the change in the pipeline’s working condition. 

**Figure 2:**
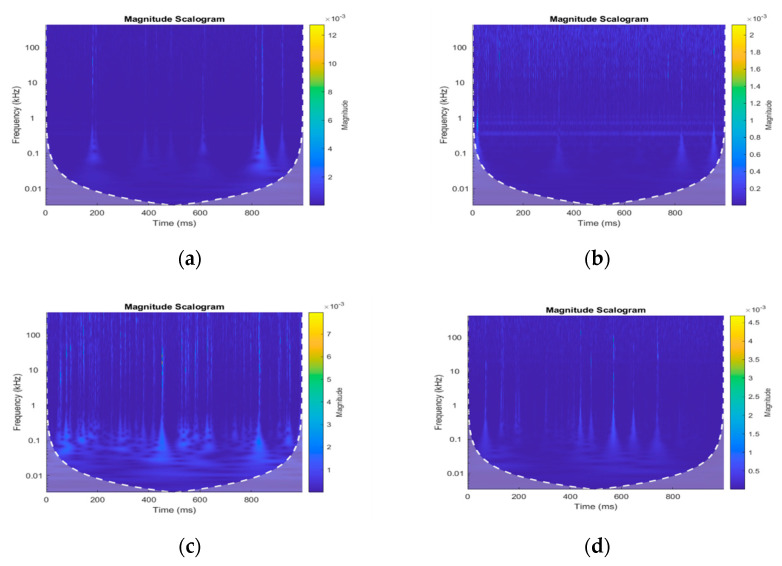
AE images: (**a**) leak condition (leak size = 0.3 mm, pressure = 7 bar); (**b**) normal conditions (pressure = 7 bar); (**c**) leak condition (leak size = 0.5 mm, pressure = 13 bar); (**d**) normal conditions (pressure = 13 bar).

In this study, a CWT with source wavelet Morse (symmetry parameter = 3) was applied to the pipeline AE signals and AE images are obtained. For complete details about Morse wavelet, readers are advised to refer to [35]. The AE images clearly show different energy regions with changing pipeline conditions as can be seen from Figure 2. As depicted in Figure 2a, when the pipeline condition changed from normal to a leak of 0.3 mm at a pressure of 2 bar, high-energy components appeared in the AE images at a different time and frequency as compared to Figure 2b. These high-energy components in the AE images at the pipeline leak conditions were due to the AE events occurring because of the leak-related stress waves. Similarly, Figure 2c shows high-energy components in the AE images that occurred due to the change in the pipeline condition from normal to a leak of 0.5 mm at a pressure of 5 bar as compared to Figure 2d. The high-energy components that appeared in the AE images at different times and frequencies was due to the AE hits generated by the leak in the pipeline. To extract the novel set of discriminant features from the AE images, the power of CNN and CAE were utilized in this study.

### 2.2. Global Feature Extraction Using CAE

#### 2.2.1. Autoencoder Background

An autoencoder consists of three layers: the input, hidden, and output layers. Together, the input and hidden layers form the encoder, while the same hidden layer combines with the output layer to make the decoder part of the autoencoder. The main purpose of the encoder network is to learn the hidden representation from the input data, whereas the decoder uses these representations to reconstruct the input data. 

The encoder part of the autoencoder can be formalized using Equation (1):

(1) $$h=a(w_{1}x+b_{1}),$$

The encoder part receives the input *x* and transforms it into latent representations, *h*, by mathematical operations in hidden layers. The weight matrix and bias vector of the bottleneck (hidden layer) are represented by *W*_1_ and *b*_1_, respectively, where *a* is the nonlinear activation function.

The encoder part of the autoencoder can be formalized using Equation (2):

(2) $$X=a(w_{2}h+b_{2}),$$

where *X* is the reconstructed data, *h* is the input of the autoencoder, and *W*_2_ and *b*_2_ are the weight and bias vectors, respectively.

While training the autoencoder, the reconstruction error between the input and output was minimized with the help of a loss function such as mean squared error. To achieve the minimum reconstruction error, the learned representation, known as latent coding, should be discriminative and of better quality. Considering the discriminative quality associated with the latent coding, in this study the autoencoder learned that latent coding were considered global features for pipeline leak state identification.

#### 2.2.2. Convolutional Autoencoder Background

The global features from the scalograms of the AE signals were extracted using a convolutional autoencoder. The CAE used the inherent property of compression in the autoencoder to extract certain useful features from which the image can be reconstructed. The CAE obtained the scalogram as an input. The encoder portion of the CAE consisted of a package of the convolutional layer followed by the pooling layer and, finally, a fully connected layer. The decoder portion of the CAE had a fully connected layer with a transposed set of convolutional layers that deconvolved the features of latent space [36]. The encoder part was used for our prime purpose, that is, features extraction, while the decoder part was needed to train the algorithm properly. Consequently, better features were learned in the shape of latent coding. Eventually, these features were transformed to reconstruct the image. The architecture of the CAE used in this study is given in Table 1.

As can be seen from Table 1, the architecture of the encoder was similar to that of the CNN presented in Table 2. With the help of convolution layers, the encoder convolved the input and took the maximum value through max pooling. The process repeated four times, and eventually the input was compressed to latent coding. In the decoder, the four transposed convolution layers (convT) upsampled the latent coding based on the stride value and then performed the convolution operation. The value of the stride for reconstruction was kept at 2 with padding equal to 0.

Equation (3) mathematically represents the convT:

(3) $$X^{m}=a^{m}(L^{m}s^{m-1}+b^{m}),$$

Here, *X^m^* is the output; *L^m^* is the convolutional matrix corresponding to layer *m*, which undergoes convolution and summation operations, transformed into vectors *s^m^*^−1^.

For the reconstruction of the input, the convolutional matrix, *L^m^*, needs to pass the transposed convolution [37]. The reconstruction process does not yield the same input but gives an output of the same dimensions. Equation (4) represents the deconvolution layer:

(4) $$Y^{m}=a^{m}({\left(L^{m}\right)}^{T}x^{m}+b^{m}),$$

Here, the output of the transposed convolution layer is denoted by *Y^m^* having the same dimensions as *S^m^*^−1^.

### 2.3. Local Feature Extraction Using CNN

The local features from the AE images were extracted using a CNN. The CNN consisted of convolutional and pooling layers that were utilized to extract the important features from the input. A fully connected layer follows the convolution and pooling layers, which perform the flattening of features and, finally, an output layer.

In the convolutional layer, several filters were used to convolve the input AE images. The AE images were 3D images; thus, the convolution process took place for each channel of the input image individually. The convolution process at each layer resulted in a feature map with the help of an activation function. The operation of the convolutional layer is expressed mathematically in Equation (5):

(5) $$x_{c}^{m}=a^{m}(\sum_{k=1}^{m-1}W_{k,c}^{m}*x_{k}^{m-1}+b_{c}^{m}),$$

In Equation (5), *m* is the number of convolutional layers, (*) depicts the 2D convolutional operation of channel *k* = 1, …, *K_m_*_−1_, at the input of the convolutional layer $x_{k}^{m-1}$, $w_{k,c}^{m}$ represents the weights of the *C*th filter in layer *m*, and *a* is a nonlinear activation function. *a^m^*(.) is used to obtain the feature map. ReLU is used as a nonlinear activation function in this operation.

In this study, max pooling was adopted for the pooling operation. The main purpose of the pooling layer was to extract useful information considering the reduction in time and memory complexity. Equation (6) shows the general expression of the feature maps obtained using the pooling layer:

(6) $$x_{c}^{m}=\beta_{c}^{m}down(x_{k}^{m-1})+b_{c}^{m},$$

Here, *down*(.) stands for the downsampling process, $x_{c}^{m}$ is the pooling layer’s output, $x_{c}^{m-1}$ is the output of the last layer and input of the current layer, and $\beta_{c}^{m}$ shows multiplicative bias, while $b_{c}^{m}$ is the additive bias.

A feature map was obtained after the data were passed through the set of convolutional and pooling layers. The feature map was further flattened by *x_m_* = *vec*(*x^m^*^−1^). The flattened feature map was passed to the fully connected layer. In a fully connected layer, the features were weighted. In this study, the fully connected layer was the output layer of the CNN. Equation (7) shows the mathematical representation of the fully connected layer:

(7) $$X^{m}=a^{m}(W^{m}x^{m-1}+b^{m}),$$

**Figure 3:**
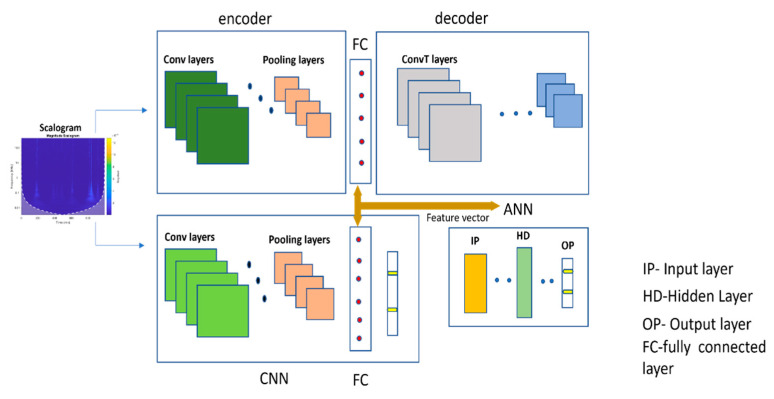
Flow diagram of local and global feature extraction and classification using an ANN.

Here, *x_m_* is the output of the fully connected layer, *a* is the activation function, and *W_m_* and *b_m_* are the weight and bias, respectively. The output of the fully connected layer of the CNN were considered as the local features in this study. The architecture of the CNN used in this paper is presented in Table 2. The local features obtained from the CNN and the global features obtained from the CAE were merged to form a discriminant feature pool. The discriminant feature pool consisted of 1024 features. Figure 3 illustrates the process of global and local feature extraction.

### 2.4. Pipeline Leak State Identification Using ANN

The ANN classifies the input features into their respective classes. The ANN has three layers: the input layer, hidden layer, and output layer. The input layer takes the features as an input for the ANN. The hidden layer linearly transforms the input features using matrix multiplication operators. The output layer performs the segregation task using an activation function. This study considered the SoftMax function as an activation function in the output layer.

Based on the input features, the ANN decides the pipeline’s condition. Here, we used a cross-categorical entropy loss function to classify the data. In this study, the ANN was used for classification; for this reason, the activation function was not applied to the input and hidden layers. The ANN can be represented using Equation (8):

(8) $$X^{m}=W^{m}x^{m-1}+b^{m},$$

Here, *X_m_* is the output of the *m*th layer, *x^m^*^−1^ is the output, and *W_m_* and *b_m_* are the weight and bias vector of the *m*th layer. Table 3 represents the architecture of the ANN used in this study.

## 3. Experimental Setup

**Figure 4:**
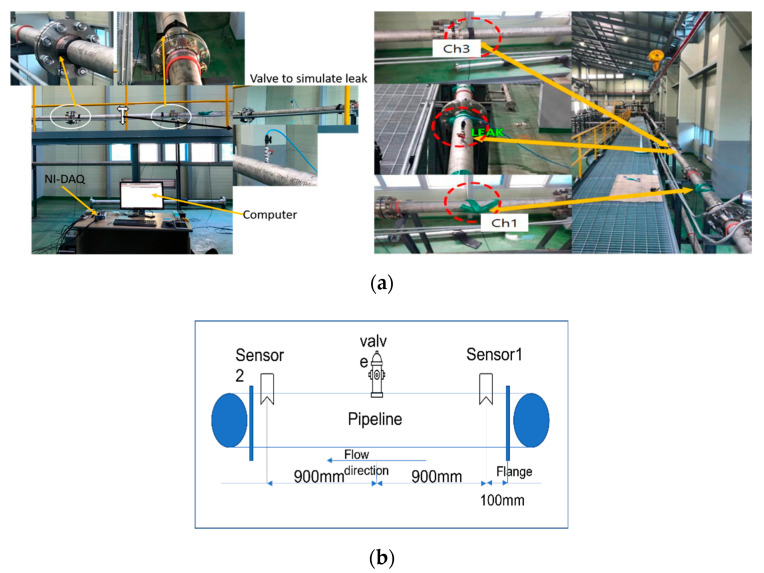
Experimental setup: (**a**) photo, and (**b**) schematic.

**Figure 5:**
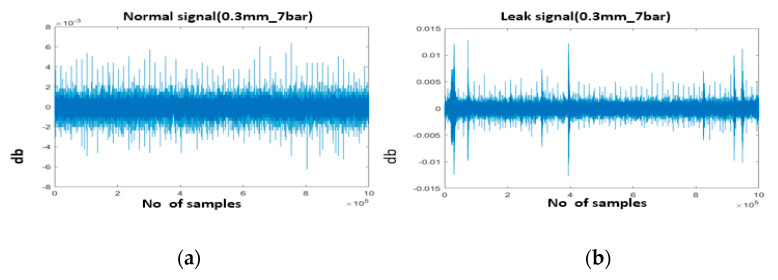

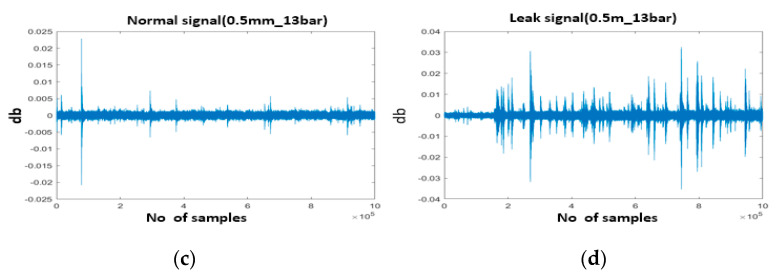
AE signals: (**a**) normal conditions (pressure = 7 bar); (**b**) leak condition (leak size = 0.3 mm, pressure = 7 bar); (**c**) normal conditions (pressure = 13 bar); (**d**) leak condition (leak size = 0.5 mm, pressure = 13 bar).

**Figure 6:**
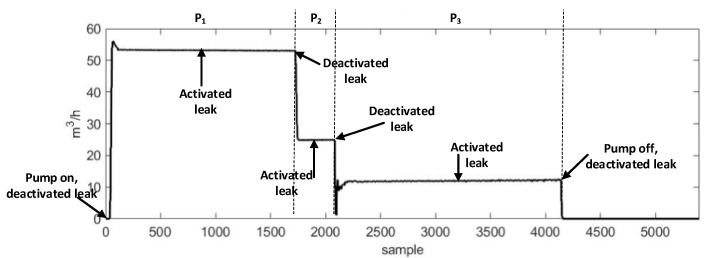
The water flow rate recorded during the experiment.

The experimental setup schematic and photos are provided in Figure 4a,b. The experimental setup consisted of a water pipeline made of stainless steel with an outer diameter of 114 mm and a thickness of 6 mm. The AE R15I-AST sensors, manufactured by Mistras Group, Inc. (New Jersey, United States) were attached to the pipeline using glue and tape. The AE signals were acquired from the pipeline at a sampling frequency of 1 MHz. To record the AE sensor data, an NI-9223 National Instruments data acquisition setup and a personal computer were used. A valve was installed on the pipeline to simulate the leak. To ensure safety, the pipeline leak valve was connected to a hose for transporting the fluid into a container, as the experiment was conducted inside the industry. The position of the valve is shown in Figure 4b. Initially, the valve was kept closed, and the pipeline was operated at normal conditions by turning on the pump. During this phase, the pressure (P1) was kept at 7 bar and data were recorded for 2 min. Then, a leak the size of 0.3 mm was simulated in the pipeline by opening the valve, and data were recorded for 2 min. After obtaining the data at 7 bar pressure, the valve was closed again and data were collected from the pipeline at 13 bar pressure (P2) for 2 min. Afterward, a leak the size of 0.5 mm was introduced into the pipeline by opening the valve (P3), and data were recorded for 2 min. Figure 5 shows the AE signals obtained from the pipeline under normal and leak conditions. Figure 6 shows the flow rate recorded during this experiment. For each pressure condition, 240 samples were collected. Of the 240 samples, 120 samples were collected under normal pipeline conditions, and the remaining 120 samples were conducted under pipeline leak conditions. 

## 4. Results and Discussion

To evaluate the performance of the proposed method, a proper testing and training data configuration is important. In this paper, leak sizes of 0.3 and 0.5 mm were created in the pipeline, and data were obtained at pressures of 7 and 13 bar. For each pressure, a total of 240 samples were obtained from the pipeline. Thus, the data set contained 480 samples for both leak sizes, out of which 240 samples were normal samples and the remaining samples were leak samples. Seventy percent of the samples were selected randomly for training purposes, and the remaining 30% of the samples were used for testing the model. To ensure the repeatability of the results, the experiments were performed ten times on each data set.

### Proposed Method: Performance and Comparison

For feature extraction, the proposed method used a combined deep neural network, CNN–CAE, which extracted both global and local features from the AE images. The global features were trends and generalized features in all the scalograms, while the local features explored the leak information from the scalograms. The local and global features were merged into a single feature vector. Finally, the feature vector was provided to the ANN for pipeline condition identification. The metrics used for comparing the proposed method against the reference method were accuracy, precision, recall, and F1 score. These metrics were calculated using Equations (9)–(12), respectively:

(9) Recall=∑αAnα×(TPαTPα+FNα)N,

(10) $$\mathrm{Pr}ecision=\frac{\sum_{\alpha }^{A}n_{\alpha }\times \left(\frac{TP_{\alpha }}{TP_{\alpha }+FP_{\alpha }}\right)}{N},$$

(11) F1=1N∑αAnα×2×∑αA(Recallα×PrecisioαRecallα+Precisioα).

(12) $$Accuracy=\frac{\sum_{\alpha }^{A}TP_{\alpha }}{N},$$

Here, $TP_{a},\;FP_{a}$, and $FN_{a}$ represent true-positive, false-positive, and false-negative results obtained from the features representing class *a*; $n_{a}$ shows all the samples from class *a*; *A* depicts the total number of classes. *N* stands for the total number of data samples in the testing sets.

**Figure 7:**
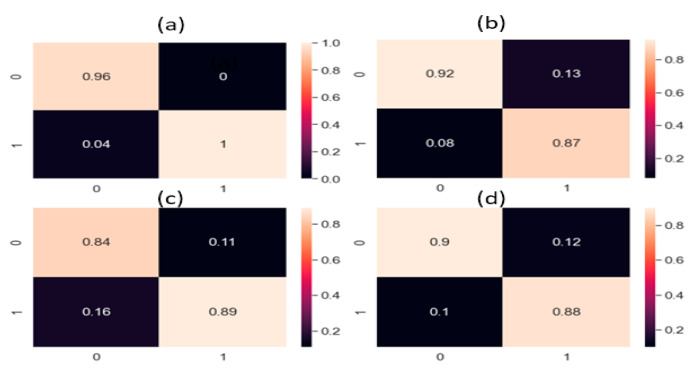
Confusion matrices of the (**a**) proposed method; (**b**) FFT-CNN; (**c**) CWT-LSTM; (**d**) CWT-SVM for the data set with a 0.3 mm leak size and 7 bar pressure.

**Figure 8:**
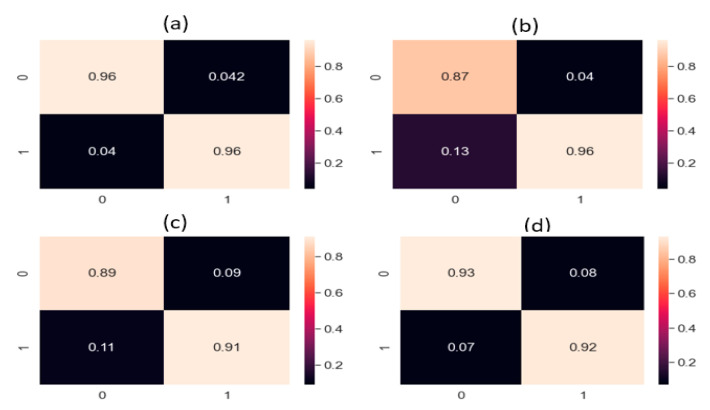
Confusion matrices of the (**a**) proposed method; (**b**) FFT-CNN; (**c**) CWT-LSTM; (**d**) CWT-SVM for the data set with a 0.5 mm leak size and 13 bar pressure.

Masoumeh Rahimi et al. [26] used a deep learning-based approach for leak detection. The data used for the study was acquired using a hydrophone from a plastic tank leak. The study compared time-domain, frequency-domain, and time–frequency domain signal pre-processing techniques, all followed by a CNN. After the signal pre-processing in multiple domains, the pre-processed signals of each domain were provided to the CNN for feature extraction and classification. The study showed that FFT-CNN performed best for leak detection. To make the comparison fair, we applied the steps provided in [26] to our pipeline data set and results were obtained. All three signal pre-processing techniques covered the features of the frequency domain or time domain but no time–frequency domain features. However, the proposed method used CWT, a time–frequency domain analysis that efficiently utilizes the change in the signal due to the presence of a leak; moreover, in the proposed technique, the AE images of CWTs were computed, which presented better-visualized features for the CNN. AE signals are noisy, non-stationary, and complex. Hence, to identify the leak-related information in changing operating conditions, discriminant features needed to be extracted. For feature extraction, the proposed method utilized a combined deep neural network, CNN–CAE, which extracted both global and local features from the AE images. The global features were trends and generalized features in all the scalograms, while the local features explored the leak information from the scalograms. The global and local features convey vital information about the leak and normal condition, even if the leak size and pressure value are changed, which is why this study used the combined neural network, CNN–CAE, for feature extraction instead of passing the raw images to traditional DL models. Although DL methods extract features, the noise in signals affect the performance of models, which is obvious from the comparisons. On the other hand, the referenced method only used a CNN to extract features and classify the data based on those features. For proving that the proposed model was superior, we compared it with two other methods: one deep learning and one machine learning method. The deep learning method was CWT-LSTM. This method takes the CWT scalogram images of the input data and feeds it to the LSTM for feature processing and classification. The machine learning method was CWT-SVM, in the same way as CWT-LSTM. It also processed the signal through CWT and computed scalograms that were fed to the SVM for identification of the leak state. Both methods gave good results but did not surpass the proposed method. The proposed method outperformed the referenced methods in leak detection because of its promising signal processing and finer-level feature extraction. Figure 7 and Figure 8 present the confusion matrices obtained from the proposed and referenced methods. The proposed model addresses the issue of leak detection at high accuracy compared to all of the referenced methods. Moreover, the proposed model was stable, because it delivered the same performance throughout the 10 experiments in this study.

The results obtained from the proposed method and the referenced methods are shown in Table 4 for both data sets (0.3 mm leak size, 7 bar fluid pressure; 0.5 mm leak size, 13 bar fluid pressure). The proposed method outperformed the referenced methods with an accuracy of 98.4%, a precision of 96.8%, recall of 97%, and an F1 score of 97.6% for the 0.3 mm and 7 bar pressure data set. Similarly, for the 0.5 mm and 13 bar pressure data set, the proposed method outperformed the reference methods with an accuracy of 96.6%, precision of 95%, recall of 95.2%, and F1 score of 95.3%. These results are explained as follows.

**Figure 9:**
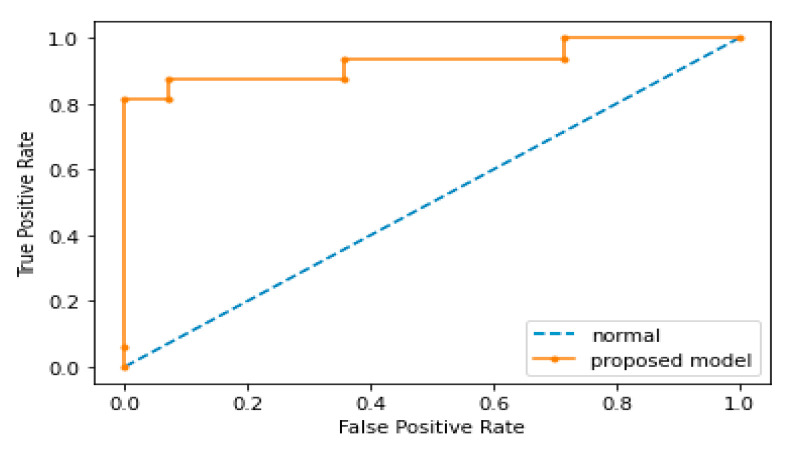
ROC curve of the proposed model.

**Figure 10:**
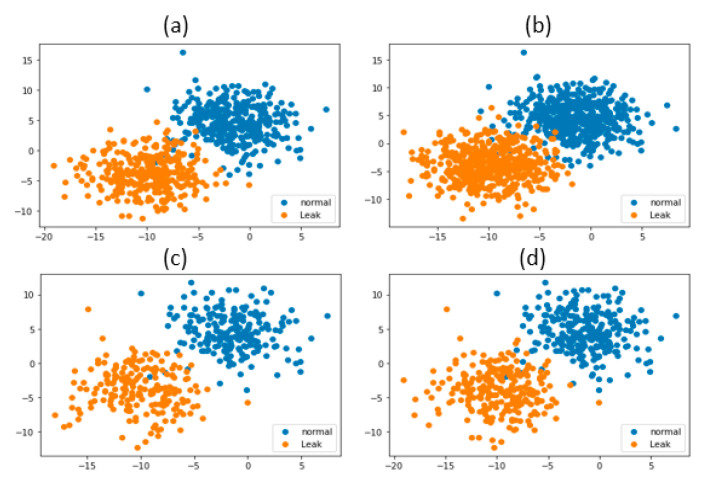
Scatter plot of the (**a**) proposed method; (**b**) FFT-CNN; (**c**) CWT-LSTM; (**d**) CWT-SVM.

The proposed model showed good classification results as well; to prove this we computed the ROC_AUC score which was 0.500 for normal conditions, while the leak condition had an ROC_AUC score of 0.9289. Figure 9 shows the ROC curve of the proposed model. For better demonstration of the comparison between the proposed and referenced models, the scatter plots were also computed. Figure 10 shows the scatter plots of the proposed method and all the referenced methods. The scatter plots were computed for the results of data set obtained from testbed having a 0.3 mm leak hole size and 7 bar fluid pressure. It is obvious from Figure 10 that the proposed method outperformed the referenced methods in terms of accuracy, since the features obtained from the proposed method were less scattered and highly discriminant for the normal and leak conditions of the pipeline. Compared to the proposed method, the features obtained from the referenced methods were either scattered or less discriminant for the normal and leak conditions of the pipeline, which is why the referenced methods underperformed in the identification of pipeline health condition. The pipeline signal was analyzed by the proposed method using MATLAB software. The total time taken for extracting the features from AE images and classification was approximately 340 s for 5 trails on a PC with a 4.2 GHz processor and a 16 GB RAM capability.

## 5. Conclusions

This paper proposed a novel deep learning-based pipeline leak detection approach. First, acoustic emission signals were collected from the pipeline under normal conditions and with different leak sizes. The acoustic emission images were obtained from the time series acoustic emission signal using continuous wavelet transform. To utilize the information in the acoustic images for pipeline leak detection, a novel deep learning framework was introduced. The novel deep learning framework was composed of a convolutional autoencoder and a convolutional neural network. The novel deep learning framework extracted a new set of local and global features from the acoustic emission images using a convolutional autoencoder and convolutional neural network. The new set of features was merged into a single feature vector. The merged feature vector was provided as input into the ANN for pipeline condition classification. In the experimental part of this study, the proposed approach was compared with a state-of-the-art method. The proposed method showed higher accuracies of 98.4% and 96.75% for different leak sizes. The proposed method can only detect leaks but cannot deliver any information about the leak localization. In the future, we will explore our proposed method in different locations of the leak with respect to the sensor and longer distances and leak localization of a pipeline.

## Figures and Tables

**Table 1:** CAE architecture.

Layers	Filters	Kernel Size	Output	Activation
Conv/MaxPool	8	3 × 3/2 × 2	128 × 128 × 8/64 × 64 × 8	ReLU/-
Conv/MaxPool	8	3 × 3/2 × 2	64 × 64 × 8/32 × 32 × 8	ReLU/-
Conv/MaxPool	8	3 × 3/2 × 2	32 × 32 × 8/16 × 16 × 8	ReLU/-
Conv/MaxPool	8	3 × 3/2 × 2	16 × 16 × 8/8 × 8 × 8	ReLU/-
Flatten	512	-	512	ReLU/-
Reshape	-	-	8 × 8 × 8	
4 covT	8	3 × 3	128 × 128 × 8	ReLU
Conv	3	3 × 3	128 × 128 × 3	ReLU

**Table 2:** CNN architecture.

Layers	Filters	Kernel Size	Output	Activation
Conv/MaxPool	8	3 × 3/2 × 2	128 × 128 × 8/64 × 64 × 8	ReLU/-
Conv/MaxPool	8	3 × 3/2 × 2	64 × 64 × 8/32 × 32 × 8	ReLU/-
Conv/MaxPool	8	3 × 3/2 × 2	32 × 32 × 8/16 × 16 × 8	ReLU/-
Conv/MaxPool	8	3 × 3/2 × 2	16 × 16 × 8/8 × 8 × 8	ReLU/-
Flatten	512	-	512	
output	4	-	4	SoftMax

**Table 3:** ANN architecture.

Layers	Nodes	Activation	Dropout Rate
Input	1024	-	0.2
Hidden	512	-	0.2
Output	2	SoftMax	-

**Table 4:** Results obtained from the proposed and reference methods.

**Metric**	**Leak Size 0.3 mm, Pressure 7 bar**			
	**Proposed**	**FFT-CNN**	**CWT-LSTM**	**CWT-SVM**
Accuracy	98.4%	96.67%	90.1%	90.33%
Precision	96.8%	96.69%	91.0%	91.0%
Recall	97%	96.6%	90.5%	91.93%
F1 Score	97.63%	96.67%	90.7%	91.53%
**Metric**	**Leak Size 0.5 mm, Pressure 13 bar**			
	**Proposed**	**FFT-CNN**	**CWT-LSTM**	**CWT-SVM**
Accuracy	96.67%	93.33%	87.67%	95.33%
Precision	95%	94.0%	85.0%	94.1%
Recall	95.27%	93.33%	88.27%	93.33%
F1 Score	95.3%	93.27%	86.3%	94.27%

## Data Availability

Data will be provided upon request.
